# Antitumor Properties of a New Macrocyclic Tetranuclear Oxidovanadium(V) Complex with 3-Methoxysalicylidenvaline Ligand

**DOI:** 10.3390/biomedicines10061217

**Published:** 2022-05-24

**Authors:** Mihaela Turtoi, Maria Anghelache, Andrei A. Patrascu, Mariana Deleanu, Geanina Voicu, Mihai Raduca, Florentina Safciuc, Ileana Manduteanu, Manuela Calin, Delia-Laura Popescu

**Affiliations:** 1 “Medical and Pharmaceutical Bionanotechnologies” Laboratory, Institute of Cellular Biology and Pathology “Nicolae Simionescu” of the Romanian Academy, 8 B.P. Hasdeu, 050568 Bucharest, Romania; maria.anghelache@icbp.ro (M.A.); geanina.voicu@icbp.ro (G.V.); florentina.safciuc@icbp.ro (F.S.); ileana.manduteanu@icbp.ro (I.M.); 2 Department of Inorganic Chemistry, Faculty of Chemistry, University of Bucharest, 23 Dumbrava Roşie, 020464 Bucharest, Romania; andrei_alunel@yahoo.com (A.A.P.); mihai.raduca@chimie.unibuc.ro (M.R.); 3 “Liquid and Gas Chromatography” Laboratory, Department of Lipidomics, Institute of Cellular Biology and Pathology “Nicolae Simionescu” of the Romanian Academy, 050568 Bucharest, Romania; mariana.deleanu@icbp.ro; 4 “C. D. Nenitzescu” Institute of Organic Chemistry of the Romanian Academy, 202B Splaiul Independentei, 060023 Bucharest, Romania

**Keywords:** oxidovanadium(V) complex, 3-methoxysalicylidenvaline ligand, hepatocarcinoma, antitumor activity, apoptosis

## Abstract

A wide variety of metal-based compounds have been obtained and studied for their antitumor activity since the intensely used cytostatic drugs (e.g., cisplatin) failed to accomplish their expected pharmacological properties. Thus, we aimed to develop a new vanadium-based drug and assess its antitumor properties using the human hepatocarcinoma (HepG2) cell line. The compound was synthesized from vanadyl sulfate, DL-valine, and *o*-vanillin and was spectrally and structurally characterized (UV-Vis, IR, CD, and single-crystal/powder-XRD). Compound stability in biological media, cell uptake, and the interaction with albumin were assessed. The mechanisms of its antitumor activity were determined compared to cisplatin by performing cytotoxicity, oxidative and mitochondrial status, DNA fragmentation, β-Tubulin synthesis investigation, and cell cycle studies. Herein, we developed a macrocyclic tetranuclear oxidovanadium(V) compound, [(V^V^O)(L)(CH_3_O)]_4_, having coordinated four Schiff base (H_2_L) ligands, 3-methoxysalicylidenvaline. We showed that [(V^V^O)(L)(CH_3_O)]_4_: (i) has pH-dependent stability in biological media, (ii) binds to albumin in a dose-dependent manner, (iii) is taken up by cells in a time-dependent way, (iv) has a higher capacity to induce cell death compared to cisplatin (IC_50_ = 6 μM vs. 10 μM), by altering the oxidative and mitochondrial status in HepG2 cells. Unlike cisplatin, which blocks the cell cycle in the S-phase, the new vanadium-based compound arrests it in S and G2/M-phase, whereas no differences in the induction of DNA fragmentation and reduction of β-Tubulin synthesis between the two were determined. Thus, the [(V^V^O)(L)(CH_3_O)]_4_ antitumor mechanism involved corroboration between the generation of oxidative species, mitochondrial dysfunction, degradation of DNA, cell cycle arrest in the S and G2/M-phase, and β-Tubulin synthesis reduction. Our studies demonstrate the potent antitumor activity of [(V^V^O)(L)(CH_3_O)]_4_ and propose it as an attractive candidate for anticancer therapy.

## 1. Introduction

Human hepatocarcinoma is the most common form of liver cancer, accounting for approximately 90% of all cancer reported cases worldwide [1]. Numerous drugs have been developed to target and treat liver cancer. Among these, cisplatin, a Pt (II) coordination compound has an important antitumor effect when is used alone or in combination with other antitumor agents (fluorouracil, doxorubicin, oxaliplatin, etc.) [2,3,4].

The pharmacological efficacy of anticancer drugs is dependent on the administration time, however, their long-term use has been shown to lead to severe side effects and cancer cell resistance to the antitumor agent [3,4]. Thus, the development of new chemotherapeutic agents with increased efficiency in the treatment of various types of cancer is necessary and urgent.

Vanadium is an essential trace element in the human body and plays various roles in some important biochemical processes [5].

Many vanadium compounds have been proven to have a wide range of biological and pharmacological effects such as antidiabetic [6], cardioprotective [7], antibacterial [8], and anticancer activities [9], and thus gained notoriety in biomedical research.

It was indicated that various vanadium complexes have higher antitumor activities than simple vanadium compounds (e.g., salts) [10]. Additionally, it was shown that the apoptotic effect of vanadocenes, organometallic compounds of vanadium(IV), is mechanistically different from the cytotoxic action of cisplatin by the fact that it is not triggered by primary DNA damage [11]. On the other hand, some studies associated the antitumor action of these organometallic compounds with the formation of vanadocene-DNA/-biomolecule adducts, still, the involved mechanism has not been clarified [12]. Different organic ligands or some molecules possessing biological properties such as amino acids-derived Schiff bases were used to develop new vanadium (IV or V) complexes with pharmacological properties [6,13,14,15,16]. The anti-tumor activity of vanadium(V) complexes with organic ligands that have the ability to chelate through donor atoms, such as O, N, O’/O, N, N’/O, O’/N, O has been reported [9,14,17,18,19]. Some of these compounds have been shown to induce reactive oxygen species (ROS)-mediated apoptosis [14]. Additionally, DNA degradation appears to be the main mechanism of the anticancer activity of up to now developed vanadium compounds [14,19]. Moreover, the vanadium complexes seem to affect the cell cycle as a result of DNA degradation and ROS generation [14,19,20]. Furthermore, the apoptosis induced in pancreatic cancer cells by the corroboration between ROS and mitochondria dysfunction was recently proposed for an oxidovanadium(IV) complex [13].

It was postulated that essentials for the pharmacological properties of the newly developed vanadium-based drugs and the rapid cell uptake are various aspects such as stability, solubility, and the capacity to bind some plasma proteins [9,14,18]. Based on the structural homology with human serum albumin and its particular intrinsic fluorescence, bovine serum albumin (BSA) is an extensively used protein in drug-protein interaction studies to estimate the drug bioavailability and transport to the specific tissues [21]. The mechanism of interaction between some vanadium compounds and BSA was widely established [6,21,22].

We aimed to develop a new vanadium-based drug with Schiff base ligand obtained from vanadyl sulfate, DL-valine, and 3-methoxysalicylaldehyde (*o*-vanillin) and then evaluate its stability in biological environments, the interaction with albumin, the cell uptake efficiency, as well as the antitumor properties against human hepatocellular carcinoma (HepG2) cell line, compared to the anticancer drug, cisplatin.

In this paper, we reported the synthesis and physico-chemical characteristics of the novel macrocyclic tetranuclear oxidovanadium(V) complex which has coordinated four Schiff base ligands, namely 3-methoxysalicylidenvaline. The newly developed vanadium-based compound (TetraV^V^) possesses a higher capacity to induce HepG2 cell death than cisplatin. Moreover, the main pathway of the antitumor action for TetraV^V^ in HepG2 cells comprises apoptosis, cell cycle arrest in the S and G2/M-phase, and β-Tubulin synthesis reduction induced by DNA fragmentation, mitochondrial dysfunction, and generation of ROS and lipid peroxidation products.

## 2. Materials and Methods

### 2.1. Chemicals and Kits

All chemicals were purchased from laboratory reagent manufacturers. Acridine orange, ammonium persulfate, bovine serum albumin (BSA), bromophenol blue, citric acid, dimethyl sulfoxide, Dulbecco’s Modified Eagle’s Medium (DMEM), ethanol, ethylenediaminetetraacetic acid (EDTA) tetrasodium salt dihydrate, Live/Dead assay kit, malonaldehyde bis-(dimethyl acetal), Mitochondrial Membrane Potential Kit, nitric acid, Nonidet P40, paraformaldehyde (PFA), perchloric acid, potassium chloride, propidium iodide (PI), RNase A, sodium orthovanadate (Na_3_VO_4_), sodium phosphate dibasic (Na_2_HPO_4_), thiobarbituric acid, trichloroacetic, Tris-HCl, Triton-X-100, 4-(2-pyridylazo)resorcinol (PAR), D-/L-valine, *o*-vanillin and vanadyl sulfate trihydrate (V^IV^OSO_4_•3H_2_O) were purchased from SIGMA-Aldrich (Merck KGaA, Darmstadt, Germany). 4′,6-Diamidine-2′-phenylindole (DAPI), fetal bovine serum (FBS), penicillin/streptomycin, 2,3-Bis-(2-Methoxy-4-Nitro-5-Sulfophenyl)-2H-Tetrazolium-5-Carboxanilide (XTT), phenazine methosulfate (PMS) and 2’,7’dichlorofluorescein diacetate (DCFH-DA) were from Thermo Fisher Scientific (Waltham, MA, USA). Cell culture dishes were from TPP^®^ (Trasadingen, Switzerland). The transparent/black 96-well micro test plates, F-bottom were from Ratiolab (Ratiolab GmbH, Dreieich, Germany) or Greiner (Greiner Bio-One GmbH, Kremsmünster, Austria) and UV 96-well micro test plates, F-bottom were from Corning Inc. (New York, NY, USA). Cisplatin was from Tocris Bioscience (Bio-Techne Ltd., Minneapolis, MN, USA). ToxiLight^TM^ BioAssay Kit from Lonza (Lonza Group AG, Basel, Switzerland), ammonium acetate from Carl Roth Gmbh (Karlsruhe, Germany), methanol Chromasolv for HPLC from Riedel (De Haen, France).

### 2.2. Synthesis of [(V^V^O)(L)(CH_3_O)]_4_ (TetraV^V^)

The new cyclic tetranuclear oxidovanadium(V) compound, [(V^V^O)(L)(CH_3_O)]_4_ has a molar ratio of 1:1 (V^V^:L). The new compound was synthesized by reacting V^IV^OSO_4_•3H_2_O (1 mmol, 0.217 g), and the Schiff base formed in a methanol solution starting from *o*-vanillin and DL-valine [6]. 1.5 mmoles DL-valine (0.176 g), 2 mmoles NaOH (0.08 g) were dissolved in 10 mL warm methanol and mixed with 1 mmol o-vanillin (0.152 g) dissolved in 5 mL methanol. The yellow Schiff base ligand was synthesized by heating the methanol solution of precursors at 60 °C for 1–2 h. The resulting dark-green mixture obtained after the reaction of the Schiff base with vanadyl sulfate hydrate was stirred at 60 °C for 2 h, followed by cooling at room temperature, and filtration. The green-brown solution was allowed at room temperature for 10–15 days and during the slow evaporation, green-brown branched crystals were formed on the glass walls. The solid compound TetraV^V^ was obtained in *cca.* 40% yield. Elemental analysis was performed, and the data are reported as %C, %H, and %V. Calc: C, 48.38; H, 5.18; N, 4.03. Found: C, 48.41; H, 5.10; N, 4.10. IR (KBr, pellets, cm^−1^): 595 ν(V – O), 625 ν(V – N), 737–801 ν_as_(V – O – CH_3_), 873 – 973ν(V = O), 1298 ν(C_Ph_ – O), 1470 ν_s_(COO^−^), 1625 ν_as_(COO^−^), 1650 ν(C = N), ≈2958 ν(C – H) methoxy/aromatic. UV-Vis (λ_max_, ε, M^−1^ × cm^−1^ for 1 × 10^−4^ M compound) in PBS, pH 7.4: 240 nm (36,184), 280 nm (31,081), 375 nm (7737).

### 2.3. Physico-Chemical Analyses

- The EuroEA Elemental Analyser system equipped with Callidus™ software was used for elemental analysis.
- A Bruker Tensor-V-37 (FT-IR) spectrophotometer was used to record the IR spectra of TetraV^V^ (KBr pellets) in the range of 4000–400 cm^−1^.
- A Jasco UV−Vis Spectrophotometer (with quartz cells of 1.0 cm path length) was used to record the electronic spectra of the 100 µM TetraV^V^ in 0.05% DMSO-phosphate buffered solution (PBS, pH = 7.4) in the wavelength range of 600–190 nm.
- A Jasco J-1500 spectrophotometer was used to study the optical activity of the 500 µM TetraV^V^ in 0.25% DMSO-PBS (pH = 7.4) by recording the circular dichroism (CD) spectra in the 500–200 nm range against DMSO-PBS.
- The STOE IPDS II diffractometer operating with Mo-Kα (λ = 0.71073 Å) X-ray tube with graphite monochromator (SHELX software) was used for elucidating the molecular structure of TetraV^V^. The molecular structure was solved by direct methods and refined by full-matrix least-squares techniques based on F2. The non-H atoms were refined with anisotropic displacement parameters. Calculations were performed using the SHELX-2013 crystallographic software package. The structures were solved by direct methods using the SHELXS structure solution program. The H atoms attached to carbon were introduced in idealized positions using the riding model. A summary of the crystallographic data and the structure refinement for crystals of TetraV^V^ are given in Table 1. CCDC reference number: 2166474.

The X-ray powder diffraction measurements were carried out on a Proto AXRD Benchtop using Cu-Kα radiation with a wavelength of 1.54059 Å in the range 5–35° (2θ).

### 2.4. BSA Binding Assay and the Stability Study in Biological Media

The capacity of TetraV^V^ (1–25 µM) to interact with albumin (2 µM BSA in PBS, pH = 7.4), was assessed by using the fluorescent quenching method described previously [6].

The absorption spectra for 100 µM TetraV^V^ in PBS (pH = 7.4) and colorless Dulbecco Modified Eagle Medium (DMEM) containing 4.5‰ glucose (pH = 7.4) supplemented with 10% fetal bovine serum (FBS) were recorded within 500 – 230 nm using the microplate reader spectrophotometer TECAN Infinite M200Pro.

The pH-dependent stability for 100 µM solution of TetraV^V^ in DMEM 4.5‰ glucose (pH = 7.4 and pH = 4.5, respectively) supplemented with 10% FBS was assessed for 24 h by registering the electronic spectra outright on the prepared solutions (designated as 0 h in the histograms), at 4 h and 24 h, respectively. The solutions read at 4 h and 24 h after preparation were maintained at 37 °C, and the recordings were performed over the entire 500–350 nm range. Data were expressed as the average ± SD of three independent measurements. Additionally, the absorbance of the compound in DMEM 4.5‰ (pH = 7.4 and pH = 4.5, respectively) was read at 405 nm and the concentration was calculated from the calibration curve of TetraV^V^ (10–200 µM).

### 2.5. Cell Culture and Drug Treatment

The human hepatocarcinoma (HepG2) cell line was purchased from American Type Culture Collection (ATCC, Manassas, VA, USA). The cells were grown in DMEM 4.5‰ glucose, supplemented with 10% FBS, 100 units/mL penicillin, and 100 µg/mL streptomycin (complete medium), as well as at 37 °C in a 5% CO_2_ incubator.

For all in vitro assays, TetraV^V^ and cisplatin were dissolved in dimethyl sulfoxide (DMSO) to make a stock solution of 100 mM concentration prepared immediately before each experiment. The working concentrations were freshly prepared in the complete culture medium in which DMSO concentration did not exceed 0.064%. HepG2 cells (1.0 × 10^5^ cells/mL) were plated for 48 h and then treated for up to 24 h with various concentrations (0.25–64 µM) of TetraV^V^ and cisplatin or with IC_50_ concentrations calculated as bellow mentioned. The cells exposed to the medium supplemented with 0.064% or 0.01% DMSO (Control) were taken under consideration.

### 2.6. Cell Death Evaluation

#### 2.6.1. Viability/Cytotoxicity Assay

For establishing the TetraV^V^ cytotoxicity compared to the antitumor drug cisplatin, three reliable assays were used.

The XTT (3-Bis-(2-Methoxy-4-Nitro-5-Sulfophenyl)-2H-Tetrazolium-5-Carboxanilide) colorimetric test was performed as previously described [23] and involves the quantification of water-soluble orange-colored formazan product released by the viable cells. Cell viability was expressed as % of control cells (100% viability). Additionally, the half-maximal inhibitory (cytotoxic) concentration for TetraV^V^ and cisplatin (IC_50_) was estimated by fitting the dose (log values)-response curves.

The ToxiLight^TM^ BioAssay Kit involves the bioluminescent quantification of the marker of cellular deterioration, adenylate kinase from the cell medium of cells treated for 4 h and 24 h with IC_50_ of TetraV^V^ (6 µM) and cisplatin (10 µM). Adenylate kinase measurement was done based on the method described previously [24]. The mean intensities for TetraV^V^ and cisplatin were normalized to DMSO exposed cells (control) and expressed as fold change.

The Live/Dead assay kit which consists of the Calcein-AM/propidium iodide (PI) staining method was also used for studying the cytotoxicity of TetraV^V^ and cisplatin at the corresponding IC_50_ concentrations. The staining protocol and the image acquisition were performed as we previously described [25]. The samples investigation was done with the 20× objective of the Inverted Microscope Olympus IX81 equipped with TRITC (λ_ex_/λ_em_ = 555 nm/580 nm) and FITC (λ_ex_/λ_em_ = 494 nm/518 nm) filters (Olympus Corporation, Tokyo, Japan). The data were expressed as dead cells (red intensity)/total cell number (red + green intensity). The final results are the average of at least 20 images/sample (≈3 fields/well). The dead/total cell number for TetraV^V^ and cisplatin was expressed as fold change to control.

#### 2.6.2. Cell Uptake and Cell Morphology Examination

TetraV^V^ internalization by HepG2 cells was investigated for 24 h by measuring the total vanadium(V) content in digested cells. HepG2 cells were treated with the IC_50_ of TetraV^V^ and 0.006% DMSO (control) for 4 h and 24 h. HepG2 cells were washed three times with PBS, detached from the culture plate, centrifuged at 3000 rpm, 4 °C for 10 min, and counted. The digestion method was adopted from Puckett C.A et al. [26]. Briefly, the cell pellet was resuspended in 65 µL of concentrated nitric acid, sonicated for 10 min in a water sonication bath, and incubated for 1.5 h, at 60 °C with constant shaking. In the end, the resulting homogeneous solution was brought up to 2 mL with water and neutralized with 10 M NaOH to reach a pH value of 6–7 (straw yellow color).

The method for vanadium(V) quantification was adapted from Such-Jen Jane Tsai and Su-Jen Hsu [27] by performing some modifications. Briefly, the digested solutions were supplemented with 1.9 mL of a chelating solution composed of 400 µL of 8 mM acetic acid-ammonium acetate buffer solution (pH = 6.0), 600 µL of 2.5 mM PAR methanolic solution, and 900 µL methanol. The resulting solutions were diluted at a 5 mL volume and incubated for 20 min at room temperature for color-developing.

The concentration of vanadium-PAR chelate in all samples was determined on a UHPLC Agilent Technologies 1290 Infinity instrument equipped with a binary pump, vacuum degasser, column oven, temperature-controlled autosampler, and diode array detector (DAD). Separation was performed on a Zorbax SB-C18 rapid RRHD reversed-phase column (2.1 × 100 mm, particle size 1.8 μm, Agilent Technologies, Santa Clara, CA, USA) maintained at 25 °C, with a flow rate of 0.25 mL/min. The mobile phase comprised the methanol-water (30/70, *v*/*v*) containing 8 mM ammonium acetate with a final pH of 6.0. The injected sample volume was 10 μL. Vanadium(V)-PAR (V^V^-PAR) chelate was detected at 540 nm. The total content of vanadium(V) in the digested cells was calculated by using a calibration curve of vanadium(V) (0.05–7.5 µg) established from a stock standard solution of 3.6 mg/mL Na_3_VO_4_ (equivalent to 1 mg/mL vanadium) and was expressed as V^V^ content (ng) contained in 10^5^ HepG2 cells. This method determines the total content of V^V^ either inside the cell or associated with the plasma cell membrane.

Data acquisition and processing were performed using Agilent ChemStation software (B.04.02 Version, Agilent Technologies).

The morphology of HepG2 cells exposed to 0.01% DMSO (Control) and HepG2 cells exposed to IC_50_ of TetraV^V^ and cisplatin for 4 h and 24 h were examined by using the 10× objective of a Zeiss microscope (Zeiss, Oberkochen, Germany).

#### 2.6.3. Oxidative Stress Evaluation

The intracellular ROS generation was detected using the oxidant-sensitive fluorescent compound, 2′,7′dichlorofluorescin diacetate (DCFH-DA). Cells treated for 4 h and 24 h with 6 µM TetraV^V^ and 10 µM cisplatin were washed twice with PBS and incubated with 20 μM DCFH-DA in PBS for 30 min at 37 °C and in the dark. Cells were washed with PBS and subjected to the membrane disintegration process in lysis buffer (40 mM KCl, 50 mM Tris-HCl, pH = 7.4, 1% Nonidet P40) on ice and to a centrifugation step at 10,000 rpm for 10 min.

The fluorescence intensity was recorded in the collected supernatant against blank cells (unexposed to DCFH-DA). Intracellular levels of ROS were expressed as DCFH-DA fluorescence intensity/µg protein and were normalized to control cells. The total protein concentration (µg/mL) in cell lysate was determined by the BCA method [28].

The extracellular lipid peroxidation products were analyzed by measuring the total malondialdehyde (MDA) content in the conditioned medium of drug-treated HepG2 cells and control cells by the TBARS method adapted from [29]. Briefly, a 500 μL medium was subsequently mixed with 300 μL of 0.1125 N perchloric acid and 300 μL of 40 M thiobarbituric acid. The resulting mixture was boiled at 97 °C for 1 h, cooled quickly at −20 °C for 20 min, and supplemented with 300 μL ethanol and 100 μL of 20% trichloroacetic acid. After short vortexing, the samples were centrifuged at 13,600× *g* for 6 min and the supernatant was transferred to a black 96-well plate. The fluorescence intensity was recorded at λ_ex_/λ_em_ = 525 nm/560 nm using the TECAN infinite M200Pro spectrophotometer. The total content of MDA in the samples was calculated by using a calibration curve of malonaldehyde bis-(dimethyl acetal) (0.16–5 µM) subjected to the same procedures as the samples. The data were expressed as a fold change of control cells.

#### 2.6.4. DNA Fragmentation Study

To study the ability of TetraV^V^ and cisplatin to induce DNA fragmentation into the treated cells, the acridine orange (AO) staining was used [25]. After drug treatment, HepG2 cells were washed three times with PBS, fixed with 4% PFA, permeabilized with 0.2% Triton X-100, and stained with AO (6 µg/mL in 0.2 M Na_2_HPO_4_, pH = 2.6/0.1 M citric acid). After 15 min, the cells were washed and stained with DAPI (1 µg/mL). The samples were examined with the 20× and 40× objectives of the Inverted Microscope Olympus IX81 equipped with TRITC, FITC, and Hoechst 33,258 (λ_ex_/λ_em_ = 345 nm/478 nm) filters. The percentage of DNA fragmentation was calculated as a ratio of red to (red + green) fluorescence subtracted from the 20× images (at least 24 images/sample, ≈3 fields/well).

#### 2.6.5. Measurement of Mitochondrial Membrane Potential (MtMP)

The MtMP was estimated using the mitochondrial-specific fluorescent dye, JC10 of the Mitochondrial Membrane Potential Kit according to the manufacturers’ instructions. HepG2 cells were plated in a black, clear bottom-96-well cell culture plate (Greiner) as mentioned above. After the treatment, the HepG2 cells were washed three times with warm PBS, and incubated with 50 µL of 1× JC10 in Buffer A for 30 min at 37 °C, in the dark. In the end, a volume of 50 µL of Buffer B was added and the fluorescence of the samples was recorded at λ_ex_/λ_em_ = 540 nm/590 nm for red-fluorescent JC-10 aggregates in the mitochondria and λ_ex_/λ_em_ = 490 nm/525 nm for green-fluorescent JC-10 monomeric form. The MtMP of drug-treated and control cells was determined by the ratio of JC-10 aggregates/JC-10 monomers and was expressed as a fold change of control cells.

#### 2.6.6. Cell Cycle Analysis

Before the treatment, HepG2 cells were synchronized in a serum-free DMEM culture medium for 12 h. Following the 24 h treatment with IC_50_ of TetraV^V^ and cisplatin, the cells were washed with PBS, detached with trypsin, and centrifuged at 2000 rpm and 4 °C for 10 min. Cells were fixed and permeabilized with 70% ethanol for 30 min on ice, centrifuged, and resuspended for 1 h in 4 µg/mL PI and 100 µg/mL RNase A in PBS. Cells were then centrifuged, washed twice in PBS, and reconstituted in FACS buffer (0.5% PFA and 1 mM EDTA in PBS). By examining the intensity of PI fluorescence with a CytoFLEX flow cytometer (Beckman Coulter; 488 nm laser, 690/50 nm Bandpass Filter, Brea, CA, USA) and analyzing the data with CytExpert 2.4.0.28 software (Beckman Coulter, Brea, CA, USA), the proportion of apoptotic cells and cell cycle phases distribution were determined. Fluorescence from at least 30,000 cells was collected.

#### 2.6.7. Immunoblotting Detection of β-Tubulin

To assess the effect of TetraV^V^ and cisplatin treatment on the expression of the microtubule’s subunit β-Tubulin, the drug-treated cells, and the control were washed twice with PBS and subjected to the lysis procedure using Radioimmunoprecipitation Assay (RIPA) buffer. All steps, including the sample preparation, electrophoretic separation, and immunological detection were performed as previously mentioned [6]. Primary antibodies: rabbit anti-β-Tubulin (1:1000, Abcam cat no. ab6046, Cambridge, UK) and mouse anti-β-actin (1:2000, Bio-Rad cat. no. MCA5775GA, Hercules, CA, USA) along with secondary antibodies conjugated with horseradish peroxidase (goat anti-mouse IgG and goat anti-rabbit IgG 1:10,000, Thermo Fisher Scientific cat no. 32430 and 32460, Waltham, MA, USA) were used for detection of target proteins. The protein expression of β-Tubulin was normalized to β-actin.

### 2.7. Statistical Analysis

Results were presented as the mean ± standard deviation (SD) of at least three independent experiments, each performed in triplicate and analyzed for statistical significance using a two-tailed Student t-test and GraphPad Prism 8 software (GraphPad Software, Version 8, San Diego, CA, USA). Statistical significance was considered for *p* < 0.05. All the fluorescence microscopy images were analyzed using Image J software (National Institutes of Health, Bethesda, MD, USA).

## 3. Results

### 3.1. Structural Studies

The crystallographic data for TetraV^V^ are presented in Table 1.

**Figure 1:**
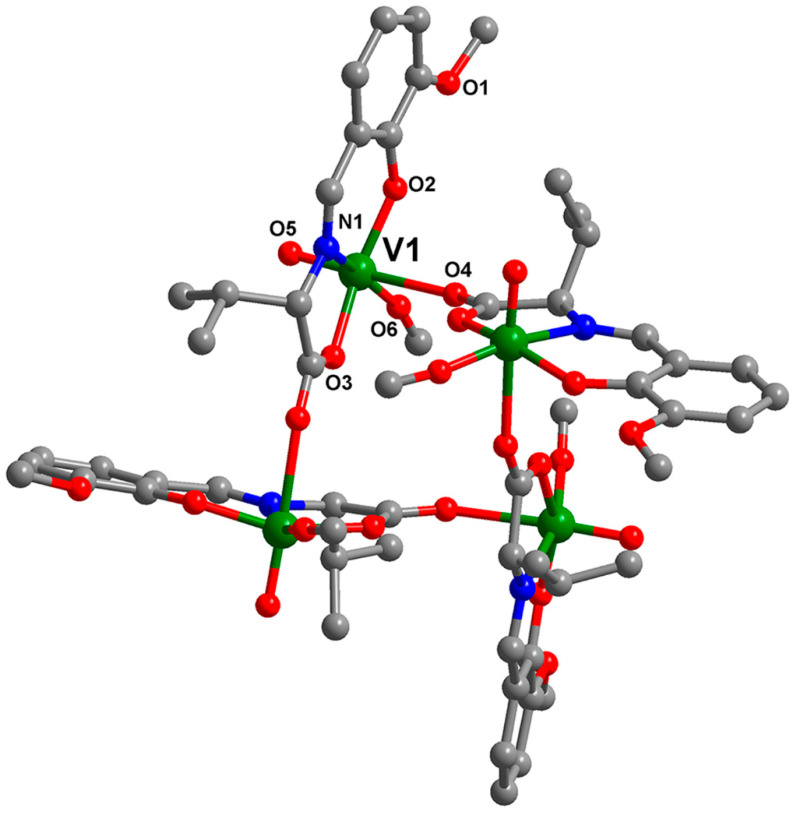
The X-ray crystal structure of TetraV^V^ with the atom labeling scheme of non-carbon atoms. For clarity, hydrogen atoms have been excluded from the diagram.

The crystal structure of TetraV^V^ consists of a neutral macrocyclic tetranuclear species (Figure 1).

The selected bond distances and angles for the TetraV^V^ complex are shown in Table S1. All vanadium atoms are equivalent, generated by symmetry, the bridge between them being assured by one carboxylic oxygen atom O4 (V – O4 = 2.290(4) Å. The vanadium metal atom presents a distorted octahedral geometry, being coordinated in the basal plane by three donor atoms from the Schiff base ligand (V – N1 = 2.120(4) Å, V – O2 = 1.856(4) Å and V – O3 = 1.947(4) Å) and one terminal oxygen atom of the methoxy anion obtain by methanol deprotonation (V – O6 = 1.750(4) Å). The apical positions are occupied by one carboxylic oxygen atom arriving from the Schiff base coordinated to another vanadium center (V – O4 = 2.290(4) Å) and one terminal oxygen atom (V = O5 = 1.567 (5) Å. Moreover, the distance established between two vanadium atoms V1 ‧‧‧ V1 is approximately 5.878 Å and is provided by the bridge formed by the oxygen atom, O4 from the composition of the Schiff base coordinated to the previous vanadium atom by O3, N1, and O2; thus, stabilizing the macrocyclic tetranuclear arrangement.

The axial bond angle (O4 – V1 – O5) and the angles formed between O4 – V1 – O6 and O4 – V1 – N1 of 177.5(3)°, 81.9(2)°, and 83.02(14)°, respectively, slightly deviate from the ideal values of 180° and 90° (Table S1). The relative arrangement of the four groups V = O in the (V^V^O)_4_ core and of the Schiff base ligands highlights a perpendicular arrangement, which prevents the ligands from overlapping and promotes the enclosure of the resulting tetranuclear structure.

**Figure 2:**
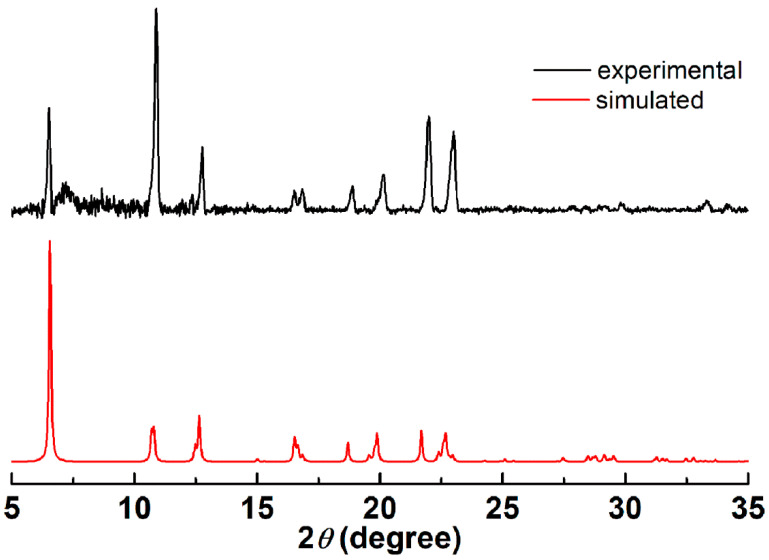
The experimental and simulated powder X-ray diffractograms for compound TetraV^V^.

The bulk sample TetraV^V^ has been analyzed also by X-ray diffraction on powder (PXRD) to ensure crystalline phase purity (Figure 2). The simulated PXRD diffractogram is in good agreement with the simulated one, the differences in peak intensities may be caused by an effect of the preferred orientation of the crystallites.

### 3.2. Spectral Studies and the Coordination Mode

#### 3.2.1. IR Spectrum

**Figure 3:**
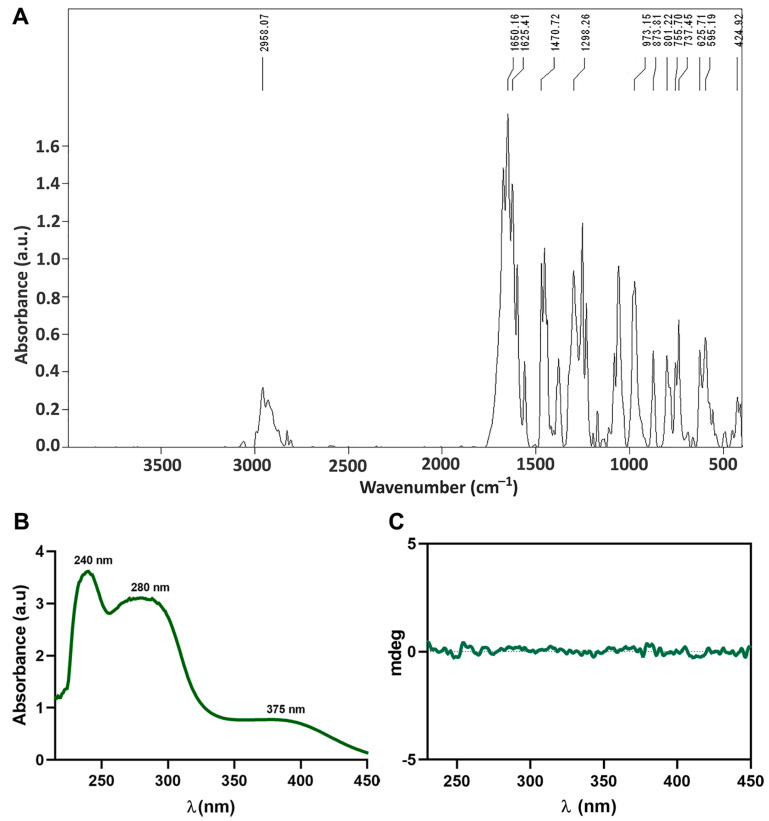
(**A**) IR spectrum of TetraV^V^. (**B**) Electronic spectrum of 100 μM TetraV^V^ in phosphate-buffered solution (PBS), pH = 7.4. (**C**) CD spectrum of 500 μM TetraV^V^ in PBS, pH = 7.4.

The infrared spectrum of the new oxidovanadium(V) compound is shown in Figure 3. The frequency assignments were done based on our previous results [6] and those of other groups [16,30]. The compound is characterized by the appearance of low-intensity absorption bands up to 3000 cm^−1^ that can be attributed to aromatic and methoxy anion C-H stretch vibration [31,32].

The absence of bands in the range of 2621–2110 cm^−1^, is associated with the frequency of the NH bond in the amino group of the DL-valine amino acid, and the displacement of the high-intensity band characteristic of the stretching frequency of the aldehyde bond (νC = O) from 1639 cm^−1^ for *o*-vanillin [6] to 1650 cm^−1^ in the case of the compound, is an indicator of the imino group formation in the Schiff base ligand and its coordination to the vanadium centers [6,16].

Additionally, TetraV^V^ shows two bands of medium-high intensity at 1625 cm^−1^ and 1470 cm^−1^ assigned to ν_as_COO^−^ and ν_s_COO^−^ with a difference between frequencies up to 200 cm^−1^ which indicates coordination of the deprotonated carboxyl group in the composition of the Schiff base in a bidentate manner with vanadium atoms [6,16]. The presence of a band of medium intensity at 1298 cm^−1^ can be associated with the vibration of the phenolic C-O bond (ν_s_C_Ph_ – O) [6]. Two bands located at 973 cm^−1^ and 873 cm^−1^ were reported to correspond to the symmetric and asymmetric stretching modes of V = O bonds and could be associated with the symmetric and asymmetric stretching modes of the V = O connection [14,33]. The medium-intensity bands between 801–737 cm^−1^ could be assigned to the vibration stretching of ν(V – OMe) [14]. Moderate to low-intensity bands appearing at 625 cm^−1^ and 595 cm^−1^ can be assigned to ν_s_V – N and ν_s_V – O (carboxyl) [6].

#### 3.2.2. Electronic Spectrum

The here developed vanadium-based drug (TetraV^V^) is a hydrophobic compound that is easily solubilized in DMSO and poorly soluble in water. Thus, to favor the solubilization of the compound in aqueous solutions (PBS or DMEM culture medium) a stock solution of TetraV^V^ in DMSO was initially prepared. The TetraV^V^ absorption spectrum was recorded for 100 µM TetraV^V^ in PBS, pH = 7.4. TetraV^V^ is characterized by 3 absorption bands with λ_max_ = 240 nm, 280 nm, and 375 nm, respectively (Figure 3B). The first two high-intensity bands characterized by λ_max_ = 240 nm and 280 nm can be associated with the π → π* transitions of the benzene ring [34] and to the imino (– CH = N –) group coordination [35]. The third medium intensity band with λ_max_ = 375 nm can be associated with the charge transfer from the oxygen in the double bond V = O to the vanadium atom (O → V) [6].

In addition, the recording of the CD spectrum for the compound TetraV^V^ (solution 500 µM TetraV^V^ in PBS) showed that it is optically inactive (Figure 3C); this is due to the coordination of both *R* and *S* enantiomers of the Schiff base ligand at the vanadium centers which makes the molecule symmetrical, a fact proven by elucidating the molecular structure.

### 3.3. BSA Binding Assay and the Stability Study in Biological Media

**Figure 4:**
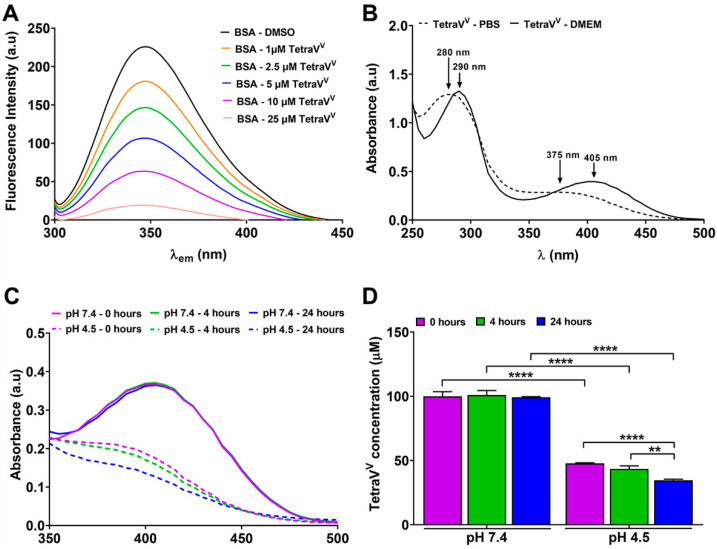
(**A**) Fluorescence spectra of 2 µM BSA in the presence of 0.1% DMSO or 1–25 µM TetraV^V^. (**B**) The electronic spectra of 100 μM TetraV^V^ in PBS, pH = 7.4 vs. DMEM culture medium, pH = 7.4. (**C**) Time dependent stability of 100 μM TetraV^V^ in DMEM medium at pH = 7.4 and pH = 4.5. (**D**) The concentration of TetraV^V^ measured at 0, 4 and 24 h in DMEM at pH = 7.4 and pH = 4.5. Data were expressed as mean ± SD: ** *p* < 0.01, **** *p* < 0.0001.

The fluorescence spectra of 2 µM BSA-PBS in the presence of DMSO or 1–25 µM TetraV^V^ highlighted the directly proportional relationship between decreasing albumin fluorescence and increasing TetraV^V^ concentration (Figure 4A), which indicates a high ability of the vanadium-based compound to interact with BSA. Using the Stern–Volmer equation and by calculating the Stern–Volmer constant from the regression line: y = ax + b (where: a = slope of the line, y = the ratio of the fluorescence intensity of free BSA solution at 347 nm to the fluorescence intensity of BSA solution at 347 nm in the presence of TetraV^V^, abbreviated I_0_/I, x = the concentration of TetraV^V^) as we previously described [6], we determined the collision extinction constant (Kq) for TetraV^V^ (Figure S1) being equal to 4.45 × e^13^ M^−1^s^−1^, indicating that TetraV^V^ interacts statically with albumin.

The comparison of the absorption spectra of TetraV^V^ (100 µM) in PBS (pH = 7.4) and the DMEM (pH = 7.4) supplemented with 10% FBS, reveals that the electronic spectrum of the TetraV^V^ is shifted to the right (to longer wavelengths) in DMEM with serum, possibly as a result of its interaction with various proteins (such as serum albumin) and growth factors contained by the cell growth medium (Figure 4B).

Furthermore, we assessed the stability of TetraV^V^ in two different biological media: (1) environment with pH = 7.4, which mimics the extracellular medium (plasma) and cytosol of cells, and (2) environment with pH = 4.5, which mimics the weakly acid medium of the lysosomes, the organelles involved in the degradation/hydrolysis of various biomolecules and extracellular components at the subunit level; thus, making them accessible to the cell. For this purpose, solutions of 100 µM TetraV^V^ were prepared in a complete DMEM culture medium with different pHs (pH = 7.4 and pH = 4.5, respectively) and incubated for 4 and 24 h at 37 °C. The absorption spectra of these solutions were recorded in the range 500–350 nm against the corresponding 0.1% DMSO solutions (Figure 4C). The concentration of TetraV^V^ was calculated for each experimental time point (the absorbance was recorded at λ_max_ = 405 nm) (Figure 4D). No changes were observed in the concentration of TetraV^V^ in DMEM at pH = 7.4 (Figure 4C,D). On the other hand, the concentration of TetraV^V^ was reduced by half (*p* < 0.001) in DMEM at pH = 4.5 compared to pH = 7.4 (Figure 4D) outright after preparation (0 h) and gradually decreased in the weakly acid medium (Figure 4D).

### 3.4. Cell Death Evaluation

#### 3.4.1. The Cytotoxic Effect of TetraV^V^ on HepG2 Cells

**Figure 5:**
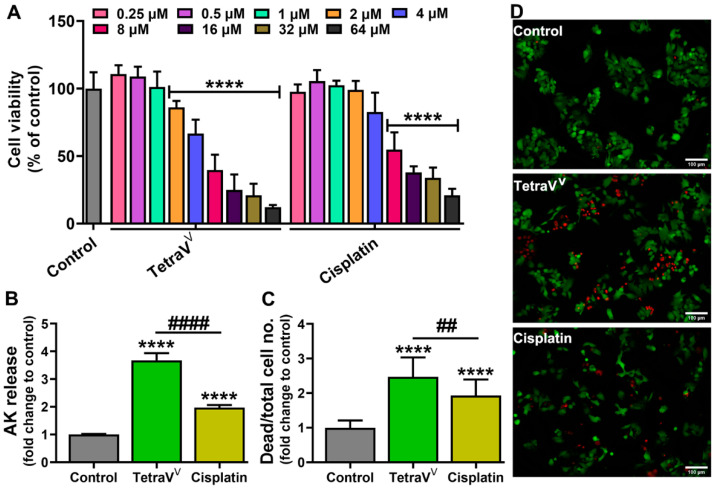
(**A**) The cell viability of HepG2 cells treated with various concentrations (0.25–64 µM) of TetraV^V^ and cisplatin. (**B**) Adenylate kinase (AK) release expressed as fold change to Control. (**C**) The number of dead cells (red fluorescence)/total cell no. (red + green fluorescence) calculated from Calcein-AM/propidium iodide (PI) staining and expressed as fold change to Control. (**D**) Representative images of calcein-AM (green)/PI (red) staining. Scale bar = 100 µm. HepG2 cells were treated with IC_50_ concentration of TetraV^V^ and cisplatin (6 µM and 10 µM, respectively) or exposed to 0.01% DMSO (vehicle, Control) for 24 h. Data were expressed as mean ± SD: **** *p* < 0.0001 vs. Control, ^##^ *p* < 0.01, ^####^ *p* < 0.0001 vs. Cisplatin.

The XTT assay showed the ability of TetraV^V^ and cisplatin to reduce the viability of liver carcinoma cells in a concentration-dependent manner compared to control cells (*p* < 0.0001) (Figure 5A). However, the concentration that induces death of half of the tested cells (IC_50_) was much lower (*p* < 0.001) in the case of TetraV^V^ (~6 µM) vs. cisplatin (~10 µM) (Figure S2A,B).

In addition, we comparatively assessed the cytotoxicity of TetraV^V^ and cisplatin at IC_50_ concentrations by two reliable tests: the ToxiLight assay which measures the released adenylate kinase (AK) in the cell media, an indicator of cellular damage, and the Live/Dead assay which is a staining method using Calcein-AM for marking viable cells in green and propidium iodide (PI) for marking dead cells in red. Thus, the exposure of the HepG2 cells to IC_50_ of TetraV^V^ and cisplatin for 24 h showed that TetraV^V^ induced a 2.6-fold increase in AK release (*p* < 0.0001) in the cell media of liver carcinoma compared to control cells, while a 2-fold increase (*p* < 0.0001) in AK release was observed for cisplatin (Figure 5B). Additionally, the cell death expressed as dead/total cell no. was increased by about 2-fold (*p* < 0.0001) in both TetraV^V^ and cisplatin-treated cells vs. control (Figure 5C,D). More than that, the AK release and the dead/total cell no. were increased by 1.9-fold and 1.3-fold, respectively, in TetraV^V^-treated cells (*p* < 0.01) vs. cisplatin (Figure 5B–D).

#### 3.4.2. Cell Uptake of TetraV^V^ and the Cell Morphology Examination

The tested drug concentration was chosen from viability/cytotoxicity studies and represents the IC_50_ of TetraV^V^ (6 µM) and cisplatin (10 µM).

The uptake of TetraV^V^ by the HepG2 cells was investigated at 4 and 24 h by determining the total content of vanadium(V) (ng) in 10^5^ cells. The quantity of V^V^-PAR chelate in all samples was determined by UHPLC. Representative chromatograms for V^V^-PAR chelate in control and cells treated for 4 and 24 h with TetraV^V^ are depicted in Figure S3.

**Figure 6:**
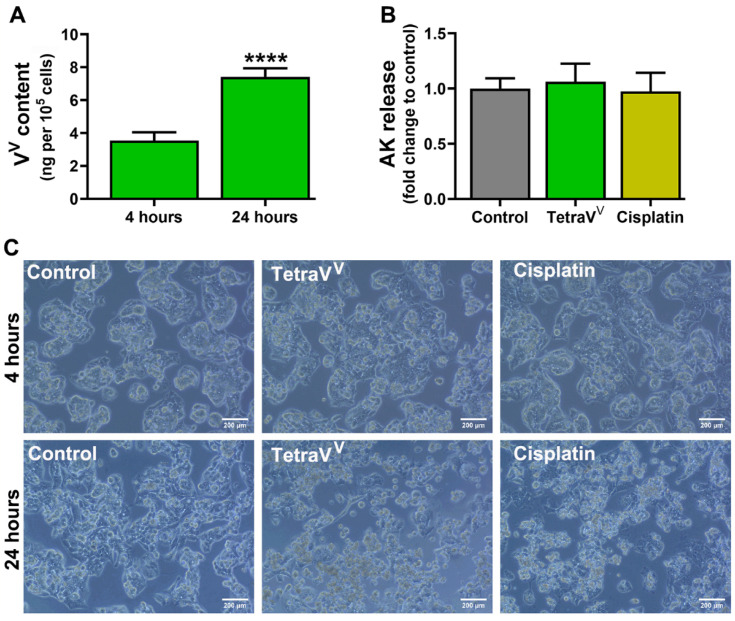
(**A**) Vanadium(V) content (ng) per 10^5^ HepG2 cells over 24 h. (**B**) Adenylate kinase (AK) release expressed as fold change to Control. (**C**) The cellular morphology of HepG2 cells exposed to TetraV^V^ and cisplatin for up to 24 h. Scale bar = 200 µm. HepG2 cells were treated with IC_50_ concentration of TetraV^V^ or cisplatin (6 µM or 10 µM, respectively) or exposed to 0.01% DMSO (vehicle, Control) for 4 and 24 h. Data were expressed as mean ± SD: **** *p* < 0.0001 vs. 4 h.

The results showed that the vanadium(V) content in HepG2 cells recorded an increase of more than 100% in 24 h compared to 4 h (*p* < 0.0001), which means that the TetraV^V^ is gradually internalized by HepG2 tumor cells over 24 h (Figure 6A).

Additionally, by performing the ToxiLight assay for HepG2 cells treated with IC_50_ concentrations of TetraV^V^ and cisplatin, we saw that both drugs had no cytotoxic effects on HepG2 cells at 4 h (Figure 6B), still, as we mentioned above, both TetraV^V^ and cisplatin increased cytotoxicity at 24 h of incubation (Figure 5) and these findings are correlated with the uptake study (Figure 6A).

Optical microscopy images for drug-treated HepG2 cells support the cell uptake and cytotoxicity studies by showing the cell phenotype changes (the cells have a rounded shape, not displayed on the plastic support) at 24 h, similar or even more pronounced to those induced by the antitumor agent, cisplatin (Figure 6C).

#### 3.4.3. The Effect of TetraV^V^ on the Oxidative Status of HepG2 Cells

To investigate the comparative effect of TetraV^V^ and cisplatin treatment on the oxidative status of HepG2 cells, the intracellular ROS and extracellular lipid peroxidation products (as total MDA levels) were determined.

**Figure 7:**
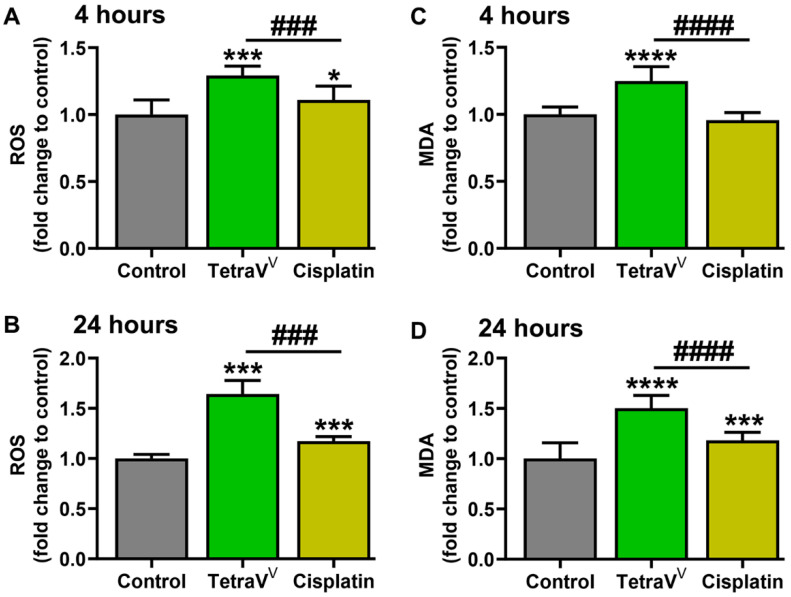
The intracellular levels of ROS expressed as DCFH-DA fluorescence intensity/µg protein (relative to control cells) in HepG2 cells at 4 h (**A**) and 24 h (**B**). The extracellular levels of MDA (relative to control cells) in HepG2 cells at 4 h (**C**) and 24 h (**D**). HepG2 cells were treated with 6 µM TetraV^V^ and 10 µM cisplatin or exposed to 0.01% DMSO (Control). Data were expressed as mean ± SD: * *p* < 0.05, *** *p* < 0.001 and **** *p* < 0.0001 vs. Control. ^###^ *p* < 0.001 and ^####^ *p* < 0.0001 vs. Cisplatin.

The results revealed a 20% to 50% increase in ROS and MDA levels in HepG2 cells treated with TetraV^V^ for 4 h and 24 h (*p* < 0.001) compared to control cells, while in HepG2 cells treated with cisplatin an ≈18% increase in both ROS and MDA levels was observed only for the 24 hours’ time incubation (*p* < 0.05) (Figure 7A–D). More than that, TetraV^V^ induced between 20% and 30% increase in ROS and MDA levels at 4 and 24 h (*p* < 0.001) compared to cisplatin (Figure 7).

#### 3.4.4. The Effect of TetraV^V^ on DNA Fragmentation and Mitochondrial Status

**Figure 8:**
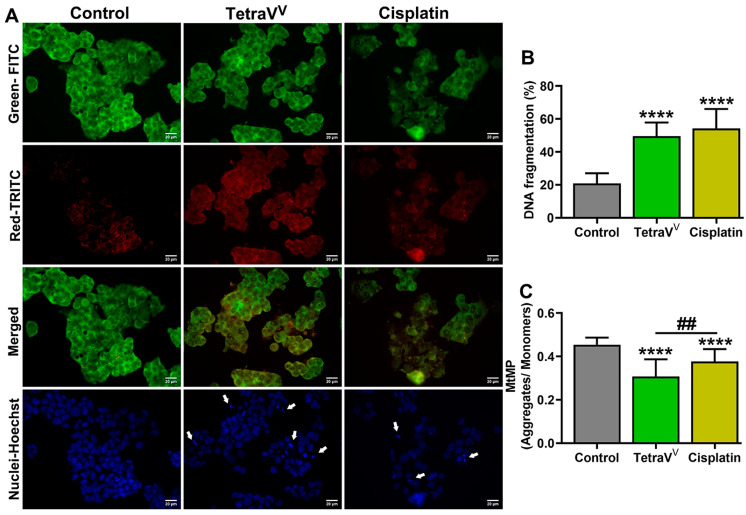
(**A**) Representative images of acridine orange and the corresponding DAPI images. (**B**) DNA fragmentation, calculated as percent of red intensity/(red+green) fluorescence. Scale bar = 20 µm. (**C**) The mitochondrial membrane potential (MtMP), expressed as the ratio of JC10 aggregates (λ_ex_/λ_em_ = 540 nm/590 nm) to JC10 monomers (λ_ex_/λ_em_ = 490 nm/525 nm). HepG2 cells were treated with IC_50_ concentrations of TetraV^V^ and cisplatin (6 µM and 10 µM, respectively) or exposed to 0.01% DMSO (Control) over 24 h. Data were expressed as mean ± SD: **** *p* < 0.0001 vs. Control. ^##^ *p* < 0.01 vs. Cisplatin. White arrows indicate the nuclear shrinkage and DNA condensation of cell nuclei.

To assess the TetraV^V^ mode of action compared to cisplatin we analyzed the DNA fragmentation and the apoptotic nuclear morphology by performing acridine orange (AO) and DAPI staining. AO is a fluorescent dye that marks in red the fragmented DNA (single-strand DNA) and in green the double-strand DNA (dsDNA). In addition, we investigated the mitochondria functionality by determining the mitochondrial membrane potential (MtMP) using the mitochondrial-specific fluorescent dye, JC10. AO/DAPI staining of tested HepG2 cells revealed that both TetraV^V^ and cisplatin increased DNA fragmentation and condensation in HepG2 cells compared to control (Figure 8A). Figure 8A reveals the typical apoptotic nuclear morphology as nuclear shrinkage and DNA condensation of cell nuclei (blue fluorescence, indicated by white arrows) and increased DNA fragmentation (red fluorescence).

The percent of DNA fragmentation calculated as a ratio of red to (red + green) fluorescence was increased by about 130% (*p* < 0.0001) in TetraV^V^ and cisplatin-treated cells vs. control (Figure 8B).

Additionally, our data showed that the cell treatment with TetraV^V^ induced a 34% decrease (*p* < 0.001) in the MtMP compared to control cells, while only a 17% (*p* < 0.01) decrease was observed for cisplatin treatment (Figure 8C). More than that, the TetraV^V^ reduced the MtMP of HepG2 cells by about 20% (*p* < 0.01) compared to cisplatin (Figure 8C).

#### 3.4.5. The Effect of TetraV^V^ on Cell Cycle and β-Tubulin Protein Expression

To investigate the comparative effect of TetraV^V^ and cisplatin treatment on the tumor cell growth we analyzed the cell cycle sequential events by flow cytometry using PI staining and the β-Tubulin protein expression.

**Figure 9:**
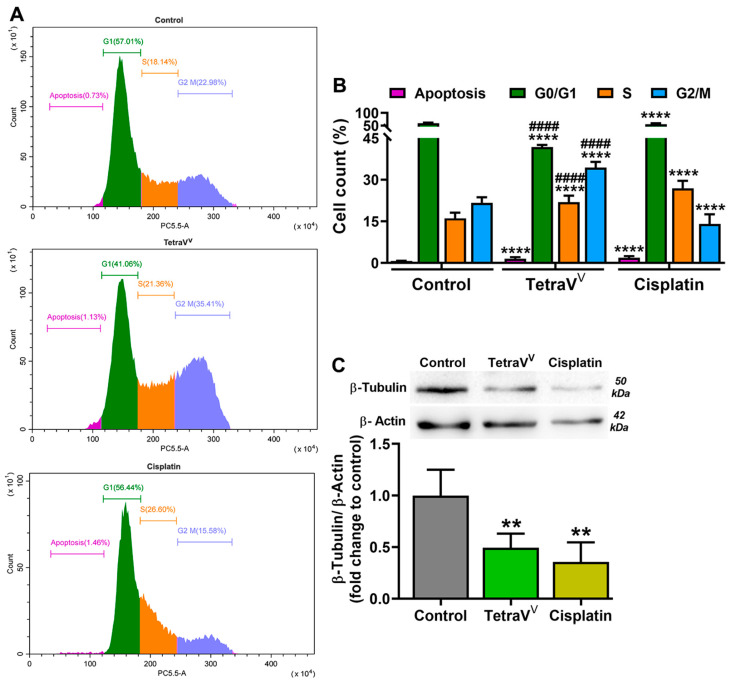
(**A**) Flow cytometry analysis of the cell cycle progression of DMSO (control), TetraV^V^, and cisplatin-treated cells. (**B**) The cell cycle phases (%) as a measure of DNA content, evaluated after propidium iodide (PI) labeling. (**C**) Determination of β-Tubulin protein expression by WB method. The representative Western blot images are depicted on top of the graph. HepG2 cells were treated with IC_50_ concentrations of TetraV^V^ and cisplatin (6 µM and 10 µM, respectively) or exposed to 0.01% DMSO (Control) for 24 h. Data were expressed as mean ± SD: ** *p* < 0.01 and **** *p* < 0.0001 vs. Control. ^####^ *p* < 0.001 vs. Cisplatin.

Our data showed that the percent population of apoptotic cells in the HepG2 cells treated with IC_50_ of TetraV^V^ (1.55 ± 0.52%, *p* < 0.0001) and cisplatin (1.88 ± 0.56%, *p* < 0.0001) was increased when compared to the control (0.69 ± 0.10%) (Figure 9A,B). The proportion of cells in G0/G1-phase decreased in both TetraV^V^ (42 ± 0.8%, *p* < 0.0001) and cisplatin-treated cells (56 ± 3.4%, *p* < 0.0001) vs. control (60 ± 1.7%), while the percent population of cells in the S-phase increased in TetraV^V^ (22 ± 2.3%, *p* < 0.0001) and cisplatin-treated cells (27 ± 2.7%, *p* < 0.0001) compared to control cells (16 ± 2.00%) (Figure 9A,B). On the other hand, a significant decrease of cells in the apoptotic, G0/G1, and S-phase (*p* < 0.001) was observed after HepG2 cell treatment with TetraV^V^ compared to cisplatin (Figure 9A,B). More than that, the corresponding percentage population of cells in G2/M-phase increased in TetraV^V^ (34 ± 2.0%, *p* < 0.0001) compared to both control (22 ± 1.9%, *p* < 0.0001) and cisplatin (14 ± 3.5%, *p* < 0.0001), while the treatment with cisplatin decreased significantly the percentage of HepG2 cells in the G2/M-phase vs. control (Figure 9A,B).

The protein expression of β-Tubulin, the subunit of microtubules, was decreased by ≈50% (*p* < 0.01) in TetraV^V^ and cisplatin-treated cells vs. control, and no significant differences were observed between TetraV^V^ and cisplatin (Figure 9C).

## 4. Discussion

Hepatocellular carcinoma is a liver cancer most often associated with various chronic liver diseases and cirrhosis, which remains one of the most prevalent cancers all over the world [25]. The use of platinum-based drugs, like cisplatin, carboplatin, or oxaliplatin in the treatment of various cancers, is well documented, however, despite their great antitumor activity, severe side effects associated with drug resistance limit their effectiveness [3,36]. Therefore, many recent studies have been carried out to develop new metal-based drugs with fewer side effects and better chemotherapeutic efficacy than platinum-based compounds [37,38].

To develop a new vanadium-based compound that possesses high antitumor activity, we used a simple method of synthesis described previously [6]. By reacting the Schiff base ligand, 3-methoxysalicylidenvaline formed in the methanol solution from *o*-vanillin and DL-valine, with vanadyl sulfate, we obtained a new macrocyclic tetranuclear oxidovanadium(V) complex which crystallized in the I4_1/a_ space group. The coordination mode of the four 3-methoxysalicylidenvaline ligands to vanadium centers, the formation of the four bridges between the carboxylic oxygen (O4) of each Schiff base coordinated to a vanadium atom and the next vanadium center, and the relative arrangement of the four groups V = O in the (V^V^O)_4_ core was indicated by spectral and structural analysis. The coordination of the O, N, O’-chelating ligands at vanadium centers with the formation of chelated rings of 5 and 6 atoms and the establishment of carboxy bridges between neighboring vanadium atoms ensure the enclosure of the tetranuclear structure and explain its stability in biological media.

Reduced drug uptake by cells, as well as other mechanisms of drug resistance, are the main obstacles to successful cancer therapy [39,40]. Essential properties for increasing the bioavailability and improving the efficiency of drug uptake by cells are compound stability in biological environments and the ability to interact with circulating proteins which may promote the drug transport to various tissues [9,14,18].

In agreement with our and other researchers’ previously published data, TetraV^V^ binds to albumin in a static mode, most likely by establishing the hydrogen bonds between the Schiff base ligands coordinated at oxidovanadium(V) centers and OH groups of the tryptophan residues from the BSA structure [6,21,22].

Moreover, our data showed that TetraV^V^ has increased stability in biological environments with physiological pH encountered in plasma and cell cytosol, a feature that ensures its uptake by HepG2 cells. A decreased stability was determined in a weakly acid medium which is most likely a result of cleavage of the interactions (by hydrolysis) established between TetraV^V^ and the transporter proteins (e.g., albumin). This result points out that the acidic pH of the lysosomes will promote the release of the TetraV^V^ after internalization and explains the biological effects observed for the newly developed vanadium-based compound. Recently, the stability of other vanadium compounds in cell culture medium at physiological pH was mentioned and these findings are following our data [14,41].

Up to date, a large number of vanadium compounds have been proposed as antiproliferative agents [9,17,18]. In this work, we analyzed the effect of TetraV^V^ on HepG2 cells viability/cytotoxicity in comparison with the standard antineoplastic agent cisplatin. The new vanadium-based compound was found to display higher cytotoxicity against HepG2 cells compared to cisplatin and this was shown also for other vanadium complexes [14]. As previously reported for another hydrophobic metallic-based compound [37], the TetraV^V^ is time-dependently uptaken by HepG2 cells, probably by both passive and active transport and this can explain the increased cytotoxic activity of TetraV^V^ at 24 h and makes it interesting for possible chemotherapeutic use. Additionally, our uptake results are in line with other studies comprising the well-known metallic antiproliferative compounds such as cisplatin, oxaliplatin [42], or the recently developed vanadium(IV) complex with a trypanostatic effect [43].

Up to now, the main proposed mechanism of action for the recently developed vanadium complexes involves ROS generation and DNA fragmentation [17,19,20]. Thus, to study the mechanism by which TetraV^V^ induces tumor cell death, we firstly assessed the oxidative status and the DNA fragmentation percent in TetraV^V^ -treated HepG2 cells compared to cisplatin. Our data revealed the time-dependent ability of TetraV^V^ to generate ROS and lipid peroxidation products compared to cisplatin which induced oxidative stress to a lower extent only at 24 h. On the other hand, similar to cisplatin, TetraV^V^ induced the damage of dsDNA. In addition, both cisplatin and TetraV^V^ altered the normal nuclear morphology to the apoptotic one, characterized by nuclear shrinkage and DNA condensation, and these findings are in good agreement with previously published studies [17,44].

The alterations of mitochondrial membrane potential (MtMP) initiate cell death in living cells [45]. It was shown that the decrease in the ratio of red-fluorescent JC aggregates in the mitochondria of cells to green-fluorescent JC monomeric form is an indicator of MtMP dysfunction and apoptosis induction [45]. The capacity of a new oxidovanadium(IV) complex to induce apoptosis by simultaneous disruption of MtMP and ROS generation was recently proved [13]. Based on these findings, we further investigated the comparative effect of TetraV^V^ and cisplatin on the mitochondrial status of HepG2 cells. In agreement with the induction of DNA fragmentation, both TetraV^V^ and cisplatin affected the mitochondrial functionality of HepG2 by reducing the MtMP, still, the TetraV^V^ induced a more pronounced mitochondrial disruption than cisplatin as a consequence of oxidative stress generation.

Cisplatin sets off cell death in HepG2 cells by apoptosis and DNA damage and by cell cycle arrest in the DNA replication/synthesis (S)-phase [46]. Our data show that, unlike cisplatin, which induces apoptosis of liver carcinoma cells by arresting the cell cycle in the S-phase, the newly developed vanadium-based compound, TetraV^V^, exerts this effect by blocking the cell cycle of HepG2 cells in both S and mitosis/cell division (G2/M)-phase. This difference between the cisplatin and TetraV^V^ mechanism of cell death induction in human hepatocellular carcinoma cells may be due to the increased properties of TetraV^V^ to generate oxidative and mitochondrial stress [13,14]. In agreement with our observations, Ni L. et al. evidenced that some multidentate oxidovanadium(IV) complexes arrested the cell cycle in the S and G2/M phases in hepatocellular carcinoma cell lines, but still the authors found a less cytotoxic effect for vanadium complexes than cisplatin [20]. In addition, other oxidovanadium(IV) complexes induced apoptosis in pancreatic cancer cells by simultaneously arresting the cell cycle in the G2/M-phase, ROS generation, and the disruption of mitochondrial membrane potential [13]. Additionally, sodium vanadate [47] and a number of vanadium(V) complexes [48] induced cell cycle arrest in the S-phase in human tumor cells. Contrary to our study, some oxidovanadium complexes were found to cause a G0/G1-phase cell cycle arrest in different types of cancer cell lines [49,50]. Cell cycle arrest is directly involved in reducing cell growth/division and together with the cellular response to DNA damage may be associated with cytoskeletal remodeling and decreased polymerization of the microtubules’ components, α- and β-Tubulin [51]. We have previously shown the involvement of cisplatin in DNA fragmentation, the cytoskeleton remodeling, and β-Tubulin synthesis reduction in human hepatocellular carcinoma cells [25]. Herein, we showed that TetraV^V^ reduced β-Tubulin synthesis after 24 h of treatment probably due to the association between ROS generation, MtMP reduction, DNA fragmentation, and cell cycle arrest in the S and G2/M-phase. However, the mechanism linking the TetraV^V^ effect with β-Tubulin synthesis is only speculative and requires future investigations. The involvement of ROS and MtMP in the destabilization of microtubules has been proposed as a mechanism for the antiproliferative effect of some drugs in breast cancer, still, the mechanisms involved are under debate [52].

Thus, our data demonstrated that apoptosis and cell cycle arrest in S and G2/M-phase, induced by DNA fragmentation, and mitochondrial membrane disruption could be one of the main pathways of the antitumor action of TetraV^V^ compound in HepG2 cells. However, other cell death pathways involving oxidative and inflammatory processes could explain the higher cytotoxic effects of TetraV^V^ compared to cisplatin. The results motivate further studies to investigate in-depth the antitumor mechanisms of the newly developed TetraV^V^ compound.

## 5. Conclusions

We developed a new macrocyclic tetranuclear oxidovanadium(V) compound with the chemical formula [(V^V^O)(L)(CH_3_O)]_4_ (TetraV^V^), where L is the deprotonated form of the Schiff base, 3-methoxysalicylidenvaline, obtained from *o*-vanillin and DL-valine, as a metal-based antitumor agent. The new compound TetraV^V^ binds the albumin in a dose-dependent manner and has pH-dependent stability in biological media, properties which ensure its internalization by the tumor cells and facilitate its release into the cell, where it exerts the biological activities. The cytotoxicity results demonstrate that TetraV^V^ has a higher antitumor effect compared to cisplatin (IC_50_ = 6 μM vs. 10 μM), the antitumor drug used in medical practice, by inducing approximately 2 times higher cell death in the liver carcinoma and also by altering the oxidative and mitochondrial status in HepG2 cells. Unlike cisplatin which blocks the cell cycle in the S-phase, the new compound arrests it in S and G2/M-phase, whereas no differences in the induction of DNA fragmentation and reduction of β-Tubulin synthesis between the two were determined. Thus, the mechanism involved in the antitumor action of TetraV^V^ comprises the induction of apoptosis, the cell cycle arrest in the S and G2/M-phase, and β-Tubulin synthesis reduction together with DNA fragmentation, mitochondrial dysfunction, and oxidative stress generation.

## Figures and Tables

**Table 1:** The crystallographic data of TetraV^V^.

Formula	C_56_H_72_N_4_O_24_V_4_	Z	4
MW (g mol^−1^)	1388.93	Calculated density (g/cm^3^)	1.458
T/K	293(2)	Absorption coefficient (cm^−1^)	0.654
λ/Å	0.71073	F(000)	2880
Crystal system	Tetragonal	Crystal size (mm × mm × mm)	0.8 × 0.5 × 0.1
Space group	I4_1_/a	θ range/deg	2.465 to 24.996
Unit cell		Limiting indices	−19 < h < 19, −19 < k < 19, −28 < l < 26
a/Å	16.377(2)	Collected reflections	29979
b/Å	16.377(2)	Sym. Indep. reflections	2793
c/Å	23.592(5)	R_int_	0.1693
α/deg	90	Data/restraints/parameters	2793/0/199
β/deg	90	GOF on F2	1.046
γ/deg	90	Final R indices	R1 = 0.0627, wR2 = 0.1431
V/Å3	6328(2)	Largest diff peak + hole/eÅ^−3^	0.535 and −0.565

## Data Availability

All data supporting reported results are included in the article.

## Supplementary Materials

The following supporting information can be downloaded at: https://www.mdpi.com/article/10.3390/biomedicines10061217/s1, Figure S1: The plot of I0/I vs. [Q] for TetraV^V^ ([(V^V^O)(L)(CH_3_O)]_4_, where L= deprotonated form of the Schiff base ligand 3-methoxysalicylidenvaline; Figure S2: (A) Nonlinear regression for % of cell viability inhibition to Log_10_ TetraV^V^/Cisplatin concentration. (B) Calculated IC_50_. Statistical significance: *** *p* < 0.001.; Figure S3: Representative chromatograms for V^V^-PAR chelate in control (A) and cells treated with TetraV^V^ for 4 (B) and 24 h (C); Table S1: Selected bond lengths (Å) and angles (°) for TetraV^V^. Symmetry operations ^#1^: −y + 5/4, x + 1/4, −z + 1/4, ^#2^: y − 1/4, −x + 5/4, −z + 1/4.

## Abbreviations

AO	Acridine orange
BSA	Bovine serum albumin
DAPI	4′,6-Diamidine-2′-phenylindole
DMEM	Dulbecco’s Modified Eagle’s Medium
DMSO	Dimethyl sulfoxide
DCFH-DA	2’,7’Dichlorofluorescein diacetate
EDTA	Ethylenediaminetetraacetic acid
FBS	Fetal bovine serum
FITC	Phalloidin-fluorescein isothiocyanate
HepG2	Human hepatocellular carcinoma cell line
H2L	3-Methoxysalicylidenvaline
MDA	Malondialdehyde
MtMP	Mitochondrial membrane potential
PAR	4-(2-Pyridylazo)resorcinol
PBS	Phosphate-buffered solution
PFA	Paraformaldehyde
PI	Propidium iodide
PMS	Phenazine methosulfate
ROS	Reactive oxygen species
Na_3_VO_4_	Sodium orthovanadate
Na_2_HPO_4_	Sodium phosphate dibasic
TetraVV	[(V^V^O)(L)(CH_3_O)]_4_
L	Deprotonated form of the Schiff base ligand
VIVOSO_4_•3H_2_O	Vanadyl sulfate trihydrate
TBARS	Thiobarbituric acid reactive substances
TRITC	Tetramethylrhodamine
XTT	2,3-Bis-(2-Methoxy-4-Nitro-5-Sulfophenyl)-2H-Tetrazolium-5-Carboxanilide
