# Supplementary material for: Antitumor Properties of a New Macrocyclic Tetranuclear Oxidovanadium(V) Complex with 3-Methoxysalicylidenvaline Ligand
Source: Biomedicines. 2022 May 24;10(6):1217. doi: 10.3390/biomedicines10061217 (PMC9220379; doi:10.3390/biomedicines10061217)
Supplement: Supplementary file 1 [file biomedicines-10-01217-s001.zip › biomedicines-1707282-Supplementary Material.pdf]

Supplementary material

Antitumor properties of a new macrocyclic tetranuclear oxidovanadium(V) complex with 3-methoxysalicylidenvaline ligand

Mihaela Turtoi [1*], Maria Anghelache [1], Andrei A. Patrascu [2], Mariana Deleanu [3], Geanina Voicu [1], Mihai Raduca [2,4], Florentina Safciuc [1], Ileana Manduteanu [1], Manuela Calin [1*], Delia-Laura Popescu [2*]

[1] "Medical and Pharmaceutical Bionanotechnologies" Laboratory, Institute of Cellular Biology and Pathology "Nicolae Simionescu" of the Romanian Academy, 8 B.P. Hasdeu, 050568-Bucharest, Romania; mihaela.carnuta@icbp.ro, maria.anghelache@icbp.ro, geanina.voicu@icbp.ro, mariana.deleanu@icbp.ro, florentina.safciuc@icbp.ro, ileana.manduteanu@icbp.ro, manuela.calin@icbp.ro.

[2] Department of Inorganic Chemistry, Faculty of Chemistry, University of Bucharest, 23 Dumbrava Roşie, 020464-Bucharest, Romania; andrei_alunel@yahoo.com, delia.popescu@chimie.unibuc.ro.

[3] "Liquid and Gas Chromatography" Laboratory, Department of Lipidomics, Institute of Cellular Biology and Pathology "Nicolae Simionescu" of the Romanian Academy, 050568-Bucharest, Romania; mariana.deleanu@icbp.ro.

[4] "C. D. Nenitzescu" Institute of Organic Chemistry of the Romanian Academy, 202B Splaiul Independentei, 060023-Bucharest, Romania; mihai.raduca@chimie.unibuc.ro. mihai.raduca@chimie.unibuc.ro,

*Corresponding authors:
M.T. mihaela.carnuta@icbp.ro
M.C. manuela.calin@icbp.ro
D-L.P. delia.popescu@chimie.unibuc.ro

Results:

Figures and Tables:

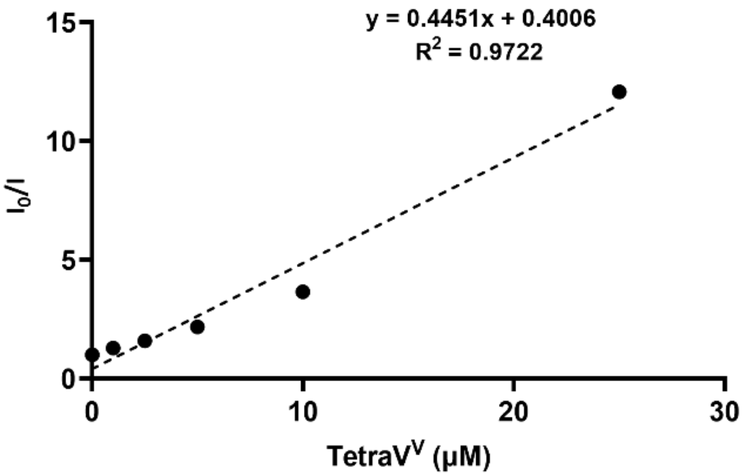

Figure S1. The plot of I_0/I vs. [Q] for TetraV^V ([(V^{V}O)(L)(CH_3O)]$_4$, where L= deprotonated form of the Schiff base ligand 3-methoxysalicylidenvaline.

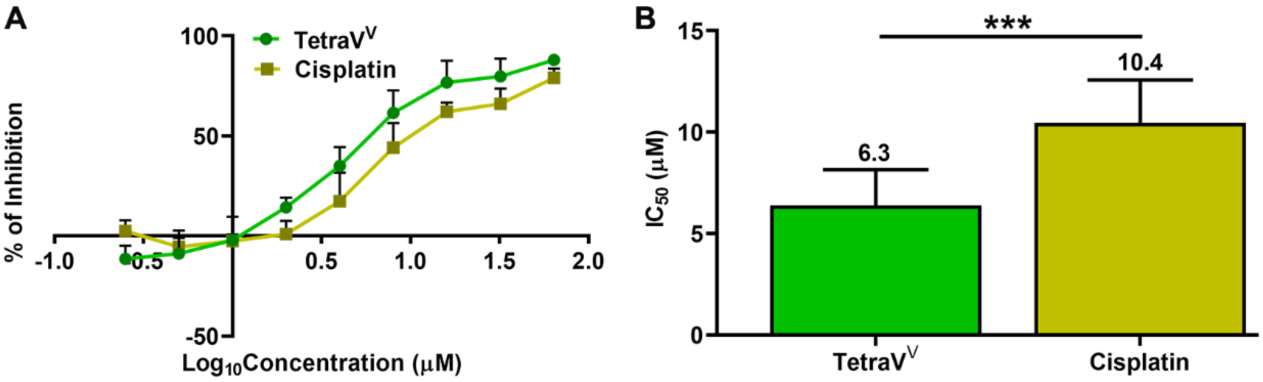

Figure S2. (**A**) Nonlinear regression for % of cell viability inhibition to Log_{10} TetraV^V/ Cisplatin concentration. (**B**) Calculated IC_{50}. Statistical significance: ***$p < 0.001$.

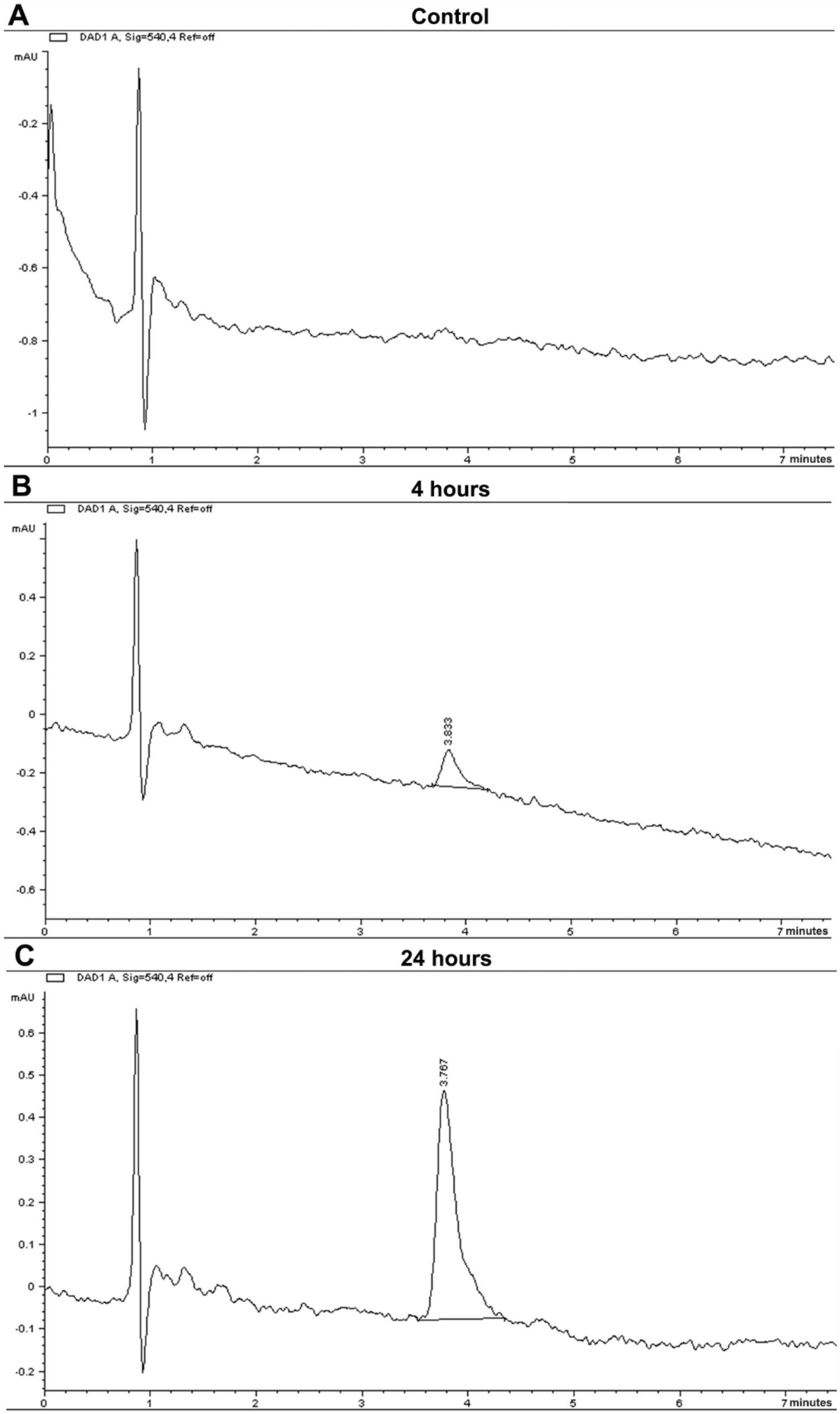

Figure S3. Representative chromatograms for V^{V}-PAR chelate in control (**A**) and cells treated with TetraV^{V} for 4 (**B**) and 24 hours (**C**).

Table S1. Selected bond lengths (Å) and angles (°) for TetraV^{V}. Symmetry operations $^{\#1}$: -y+5/4, x+1/4, -z+1/4, $^{\#2}$: y-1/4, -x+5/4, -z+1/4.

**Bond**	(Å)	**Angle**	(°)	**Angle**	(°)
N(1) – V(1)	2.120(4)	O(2) - V(1) - O(3)	156.81(16)	O(3) - V(1) - N(1)	76.79(15)
O(2) – V(1)	1.856(4)	O(2) - V(1) - O(4)$^{\#2}$	80.37(17)	O(4)#2 - V(1) - O(5)	177.5(3)
O(3) – V(1)	1.947(4)	O(2) - V(1) - O(5)	97.9(3)	O(4)#2 - V(1) - O(6)	81.9(2)
O(4) –V(1)$^{\#1}$	2.290(4)	O(2) - V(1) - O(6)	99.48(19)	O(4)#2 - V(1) - N(1)	83.02(14)
O(5) – V(1)	1.567(5)	O(2) - V(1) - N(1)	85.77(16)	O(5) - V(1) - O(6)	100.2(3)
O(6) – V(1)	1.750(4)	O(3) - V(1) - O(4)$^{\#2}$	82.43(16)	O(5) - V(1) - N(1)	95.1(2)
		O(3) - V(1) - O(5)	98.8(2)	O(6) - V(1) - N(1)	163.0(2)
$^{\#1}$: -y+5/4, x+1/4, -z+1/4		O(3) - V(1) - O(6)	93.34(18)	$^{\#2}$: y-1/4, -x+5/4, -z+1/4	
